# Supplementary material for: Characterization of a Salmonella enterica serovar Typhimurium lineage with rough colony morphology and multidrug resistance
Source: Nat Commun. 2024 Jul 20;15:6123. doi: 10.1038/s41467-024-50331-y (PMC11271444; doi:10.1038/s41467-024-50331-y)
Supplement: Supplementary file 1 — Supplementary Information [file 41467_2024_50331_MOESM1_ESM.pdf]

Contents

SUPPLEMENTARY FIGURES **2**
Figure S1. Biofilm formation by the representative rough and smooth colony strains. 2
Figure S2. Examination of the red, dry, and rough morphotype and pellicle formation in representative rough and smooth colony strains. 3
Figure S3. Biofilm production by representative rough and smooth colony strains determined by confocal laser scanning microscopy (CLSM) at 28 °C. 4
Figure S4. Minimum spanning tree comparing core-genome allelic profiles among different groups (rough type, smooth type, and other). 5
Figure S5. Distribution of the 3,951 *S.* Typhimurium isolates among different lineages. 6
Figure S6. Bayesian evolutionary analysis showing the maximum-clade credibility tree. 7
Figure S7. Box plots representing the antimicrobial resistance genes and virulence factors among different phylogenetic lineages and colony phenotypes. 8
Figure S8. Maximum likelihood phylogeny of the global *S.* Typhimurium isolates with a heatmap showing the distribution of the antimicrobial-resistant determinants. 9
Figure S9. Maximum likelihood phylogeny of the global *S.* Typhimurium isolates with a heatmap showing the distribution of virulence factors. 10
Figure S10. Pangenome analysis of the global *S.* Typhimurium isolates. 11
Figure S11. Accessory genes identified by pangenome analysis that might be associated with rough colony variants. 12
Figure S12. The SNPs identified by GWAS analysis that might be associated with rough colony variants. 13
Figure S13. Proteomics analysis of the representative rough and smooth colony strains. 14
Figure S14. Growth of SF175 and SF175-Δ*rpoS* on different media. 15
SUPPLEMENTARY METHODS **16**
Congo red assay 16
Pellicle formation 16
Scanning electron microscopy (SEM) examination 16
Crystal violet biofilm assays 17
Confocal laser scanning microscopy (CLSM) imaging 18
Whole-genome sequencing and genome assembly 19
Single nucleotide polymorphism (SNP) calling and phylogenetic analysis 19
Plasmid detection 20
Genome-wide association study (GWAS) 21
Recombination analysis 21
Biofilm-related genes and mutation detection 21
Proteomic analysis of the rough and smooth colony strains 22
Construction of the mutants with *csgD* gene knockout and a single nucleotide replacement (-44 T>G) in its promoter 26
SUPPLEMENTARY REFERENCES **28**

SUPPLEMENTARY FIGURES

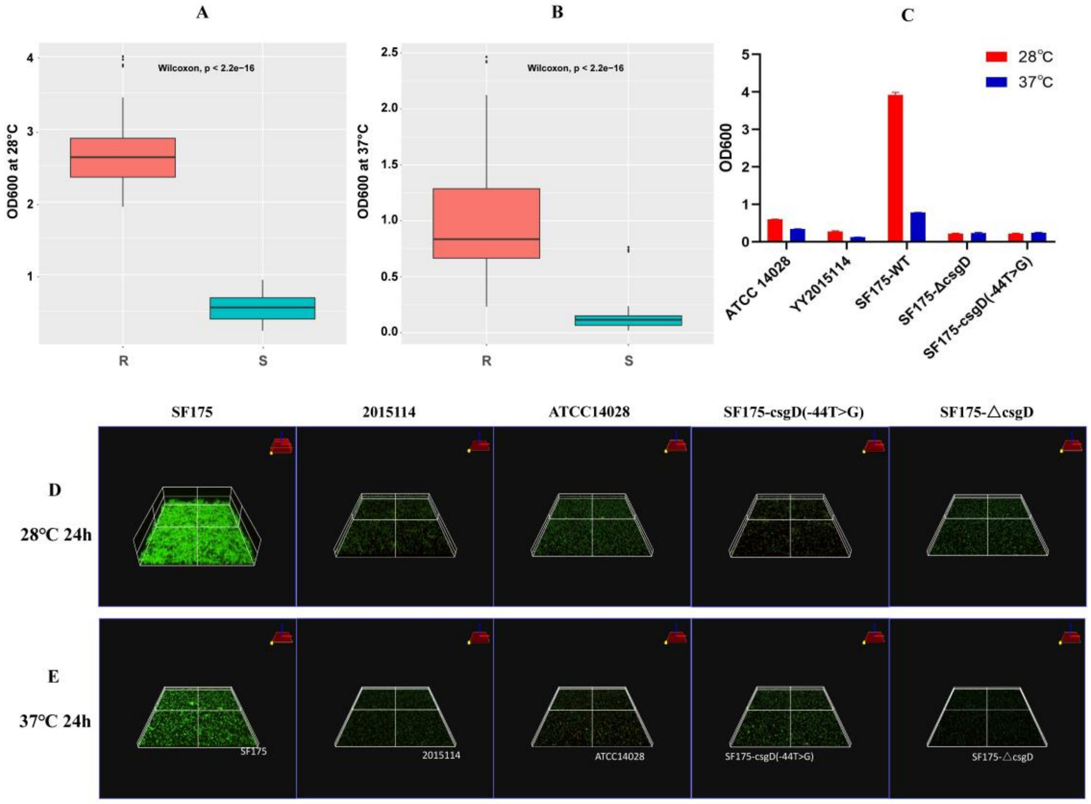

Figure S1. Biofilm formation by the representative rough and smooth colony strains.

A and **B**. Biofilm production by the selected 20 rough and 20 smooth colony strains determined by crystal violet assays at 28 °C and 37 °C. The assay is representative of three independent experiments carried out in triplicate. Data are expressed as a box plot. Centerline, median; box limits, upper and lower quartiles; whiskers, 1.5x interquartile range. **C**. Biofilm production by the representative *S*. Typhimurium isolates including the rough colony strain SF175, smooth colony strain 2015114, control strain ATCC 14028, and mutants SF175-Δ*csgD* and SF175-csgD with -44T>G determined by crystal violet assays at 28 °C and 37 °C. Error bars represent standard deviations. **D** and **E**. Biofilm production by the representative *S*. Typhimurium isolates determined by CLSM at 28 °C and 37 °C. The strains were grown for 24 hours and then stained with PI (30 mg/mL, red fluorescence, labeled bacterial cells) and FITC-ConA (10 mg/L, green fluorescence, labeled extracellular polymeric substance), and 3D images were acquired by CLSM. Source data are provided as a Source Data file. R, rough; S, smooth; CLSM, confocal laser scanning microscopy.

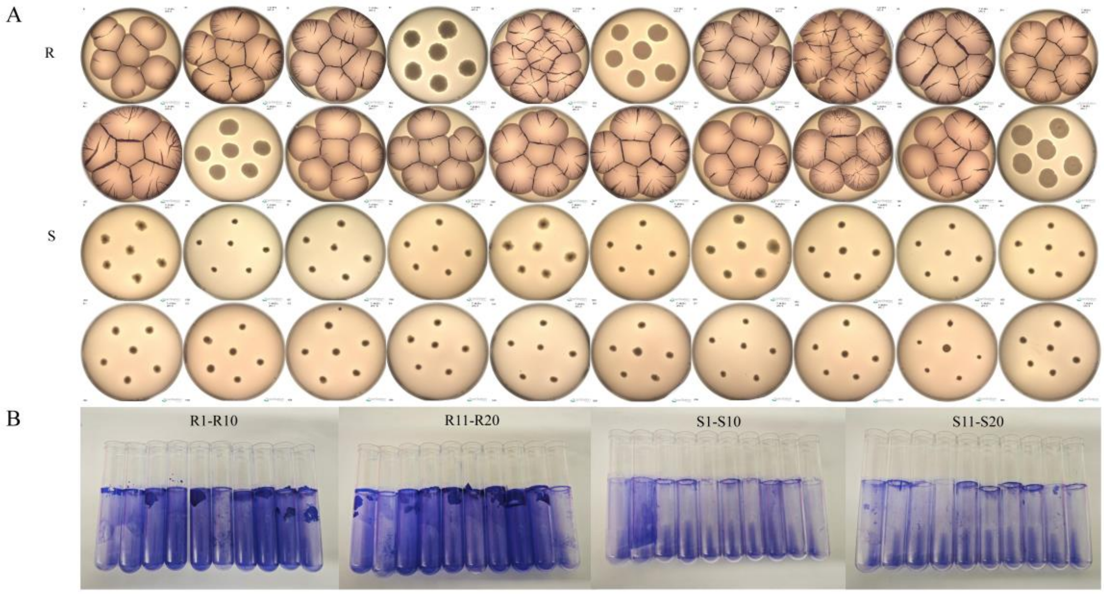

Figure S2. Examination of the red, dry, and rough morphotype and pellicle formation in representative rough and smooth colony strains.

A. The ability of 20 representative rough colony strains and 20 representative smooth colony strains to exhibit the rdar phenotype on media containing Congo red, incubated at 37 °C for 48 hours. **B.** Pellicle formation at the liquid-air interface of rough and smooth colony strains after staining with crystal violet. Source data are provided as a Source Data file. R, rough; S, smooth.

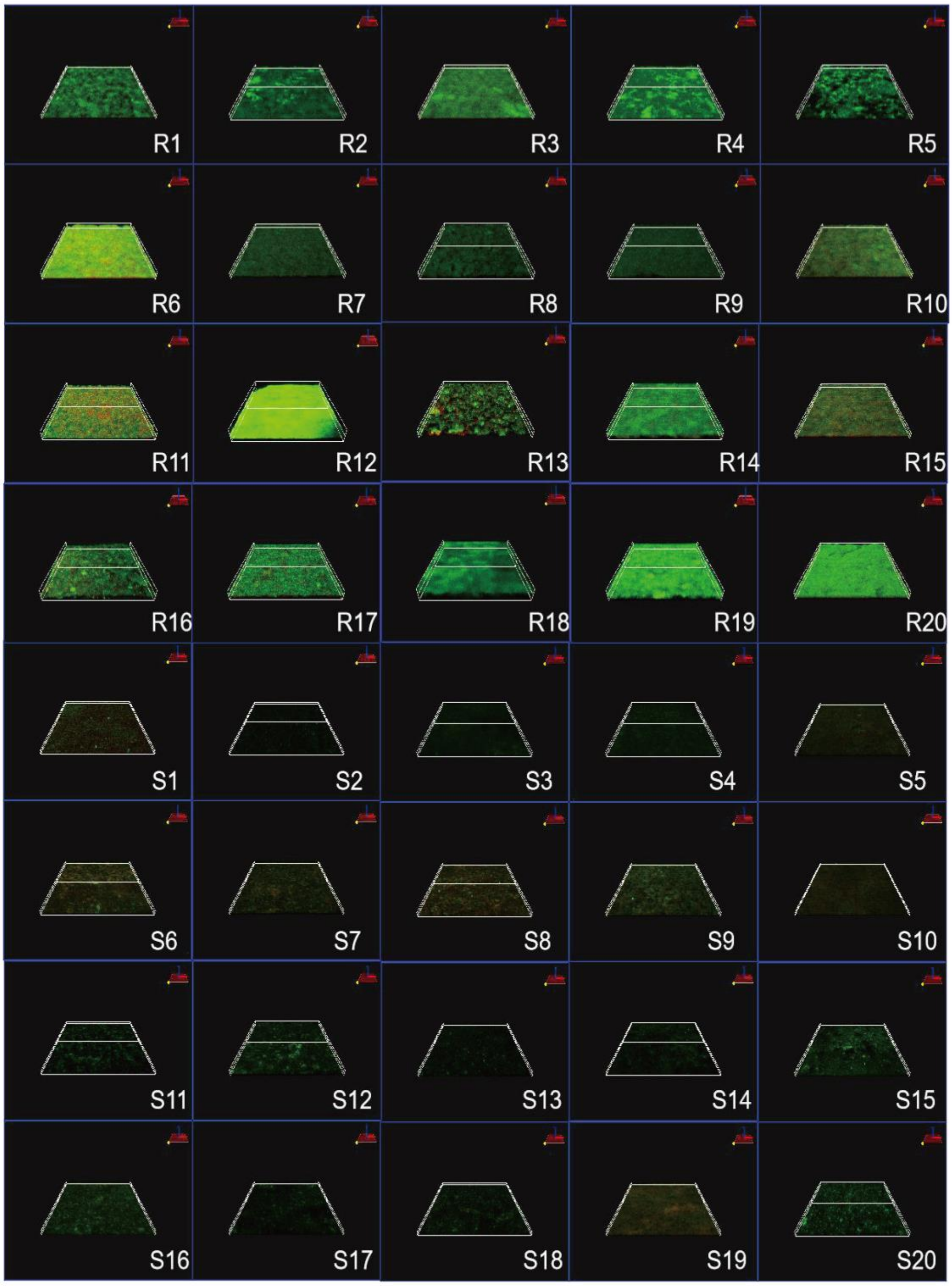

Figure S3. Biofilm production by representative rough and smooth colony strains determined by confocal laser scanning microscopy (CLSM) at 28 °C.

Source data are provided as a Source Data file. R, rough; S, smooth.

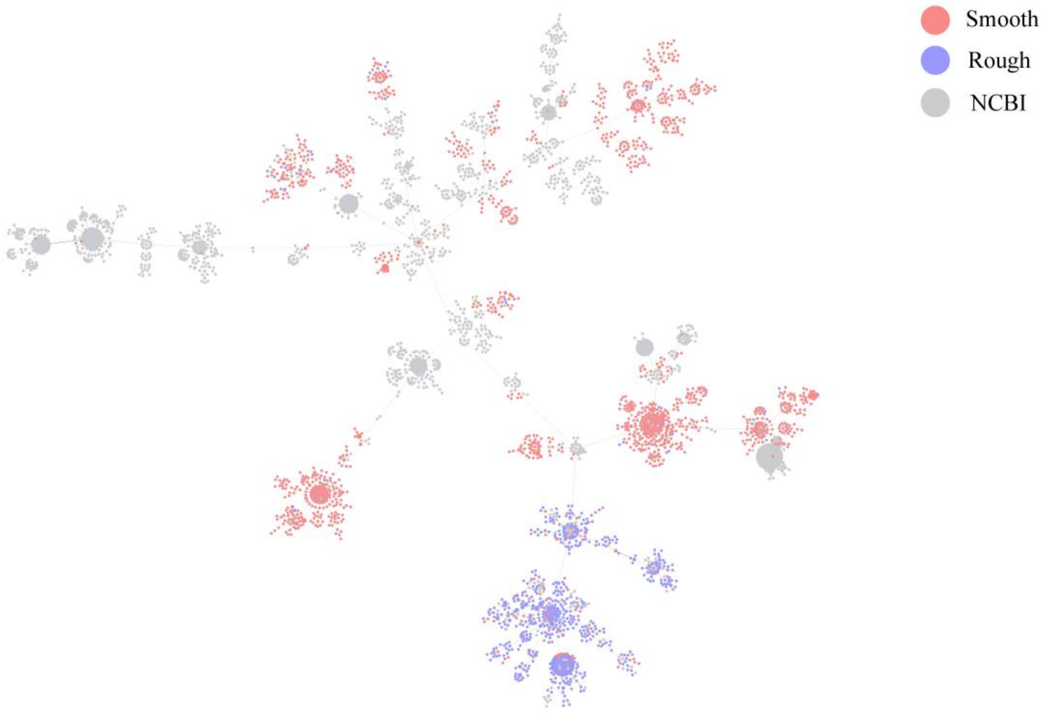

Figure S4. Minimum spanning tree comparing core-genome allelic profiles among different groups (rough type, smooth type, and other).

Source data are provided as a Source Data file.

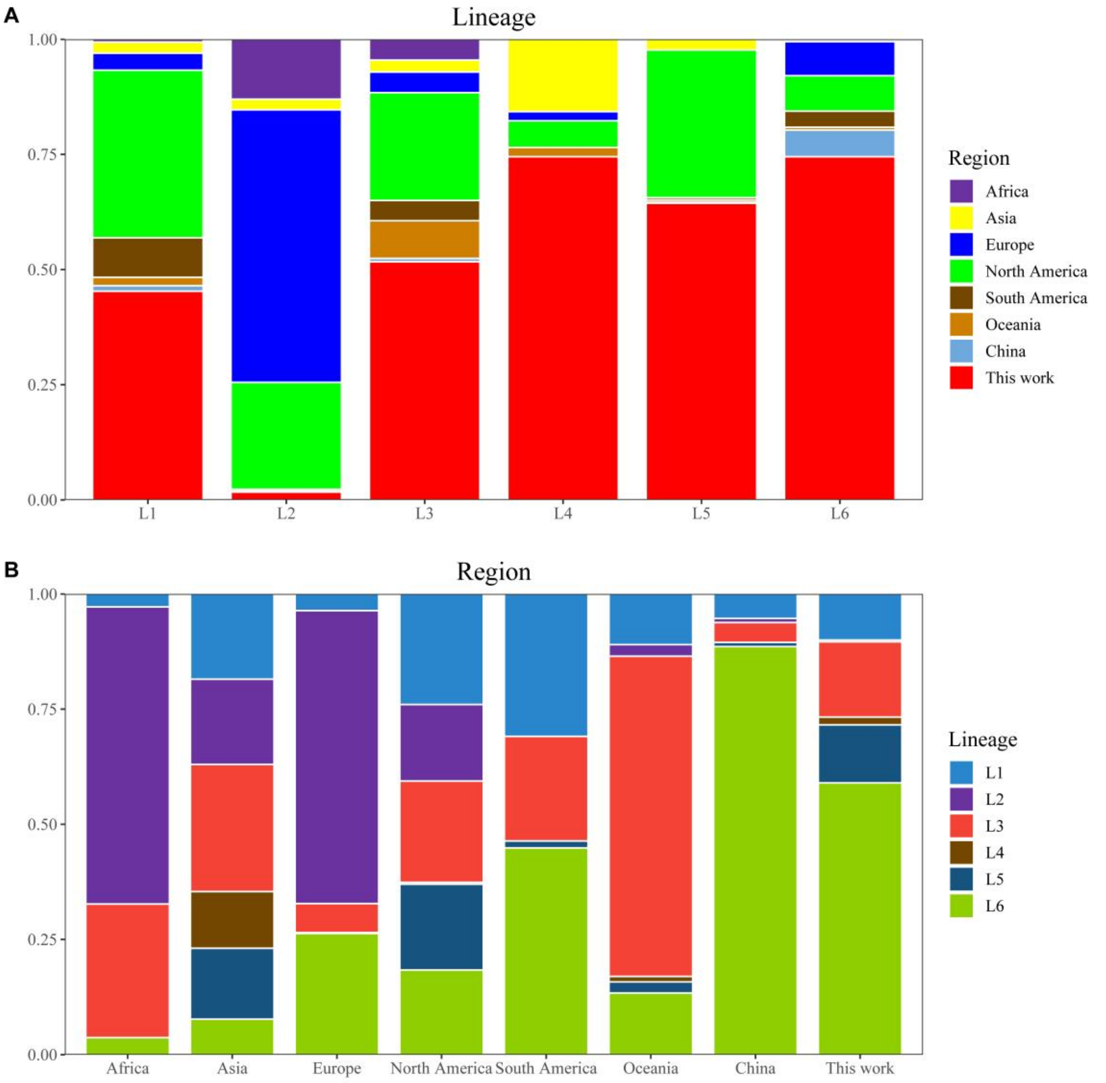

Figure S5. Distribution of the 3,951 *S*. Typhimurium isolates among different lineages.

A. Geographic distribution of the *S*. Typhimurium isolates in each genetic lineage. **B**. Lineage distribution of the *S*. Typhimurium isolates in each geographic region. Source data are provided as a Source Data file.

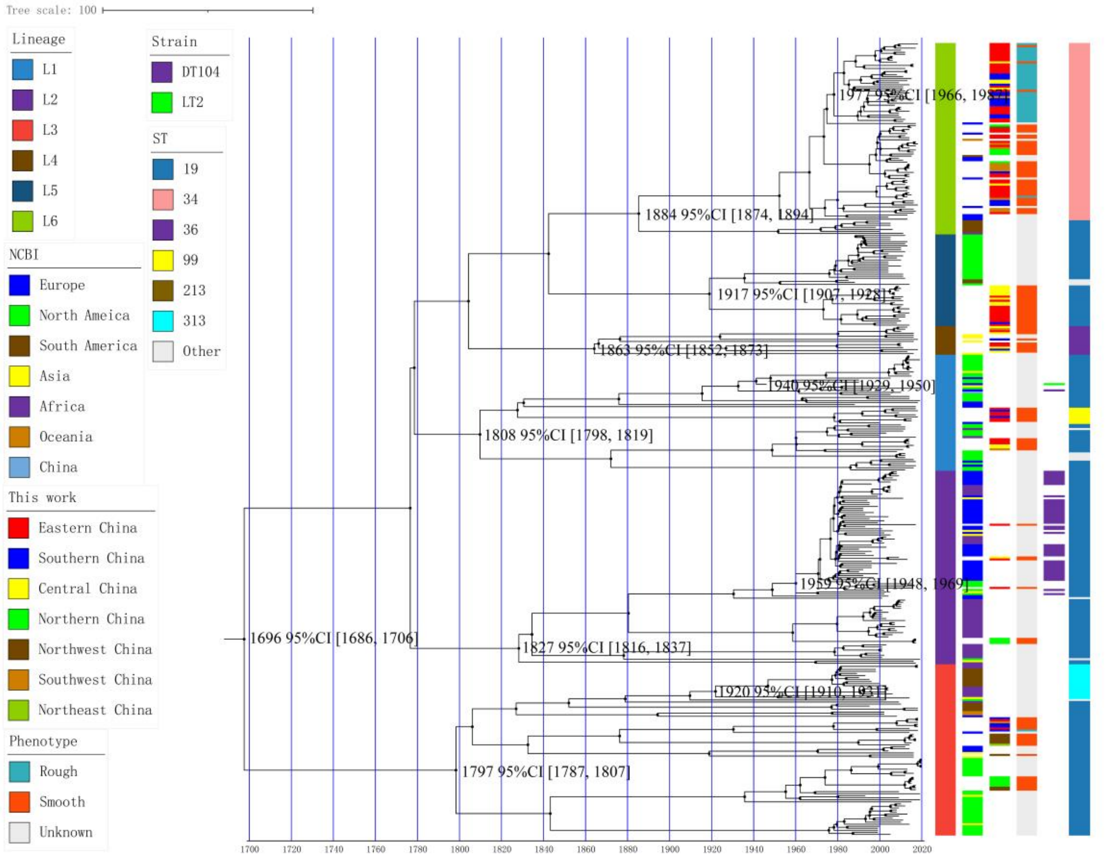

Figure S6. Bayesian evolutionary analysis showing the maximum-clade credibility tree.

The maximum-clade credibility tree was inferred from 12,179 SNPs from 401 representative strains with definite isolation times and regions. The most recent common ancestor of *S.* Typhimurium DT104 was predicted in this study to date to 1959 (95% confidence interval: 1948 to 1969), consistent with a previous study[1]. Source data are provided as a Source Data file. ST, sequence type; SNP, single nucleotide polymorphism.

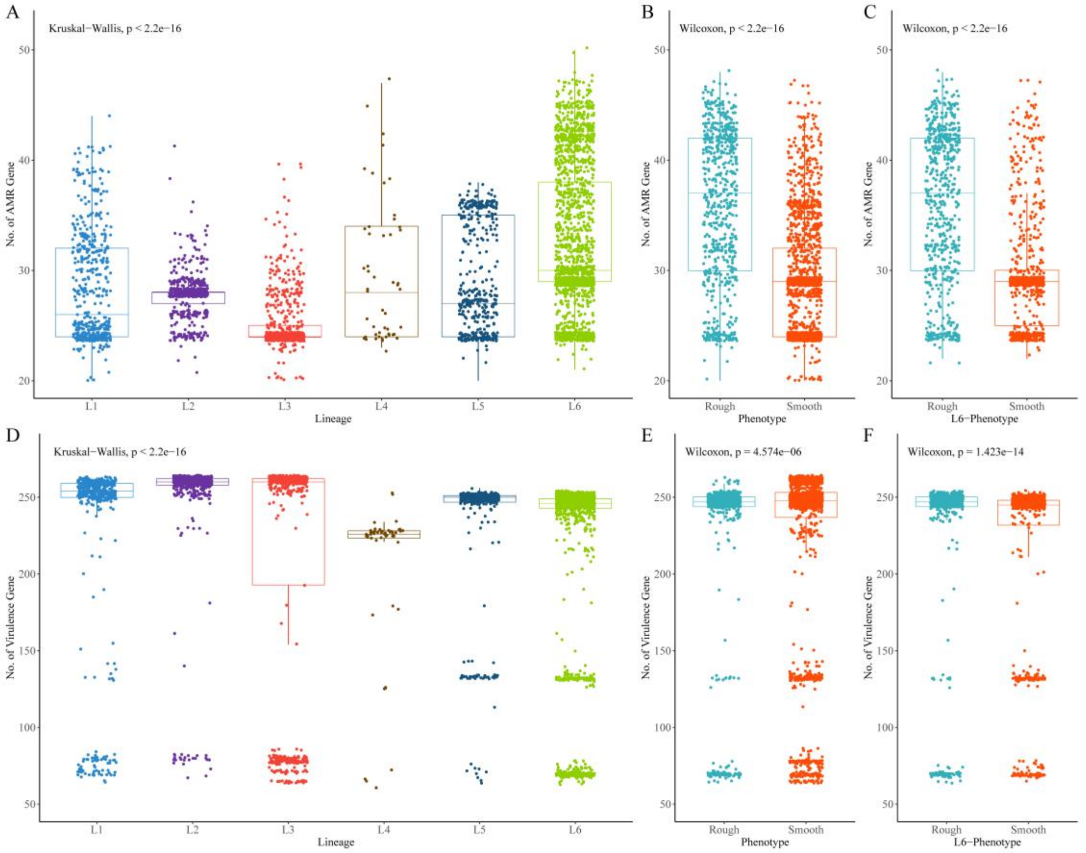

Figure S7. Box plots representing the antimicrobial resistance genes and virulence factors among different phylogenetic lineages and colony phenotypes.

A, **B**, and **C** show the number of antimicrobial resistance genes among the different lineages, colony phenotypes, and colony phenotypes in Lineage 6, respectively. **D**, **E**, and **F** show the number of virulence factor genes among the different lineages, colony phenotypes, and colony phenotypes in Lineage 6, respectively. Centerline, median; box limits, upper and lower quartiles; whiskers, 1.5x interquartile range; points, individual data. Source data are provided as a Source Data file. AMR, antimicrobial resistance.

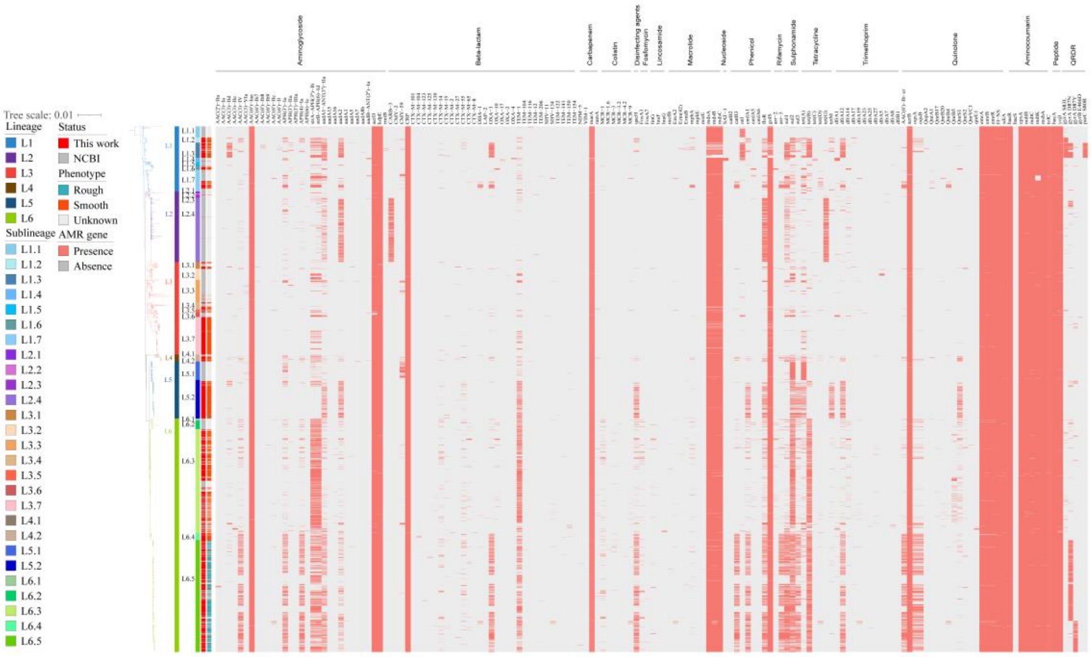

Figure S8. Maximum likelihood phylogeny of the global *S*. Typhimurium isolates with a heatmap showing the distribution of the antimicrobial-resistant determinants.

The bands to the left of the heatmap indicate the lineage, sublineage, source, and colony morphology of the strains. The presence of an antimicrobial resistance gene is indicated in red, and the absence of a gene is indicated in gray. The QRDR mutations, including S83L, D87N, and D87Y in the *gyrA* gene, E466D in the *gyrB* gene, and S80R in the *parC* gene, are indicated separately. Source data are provided as a Source Data file. AMR, antimicrobial resistance; QRDR, quinolone resistance-determining region.

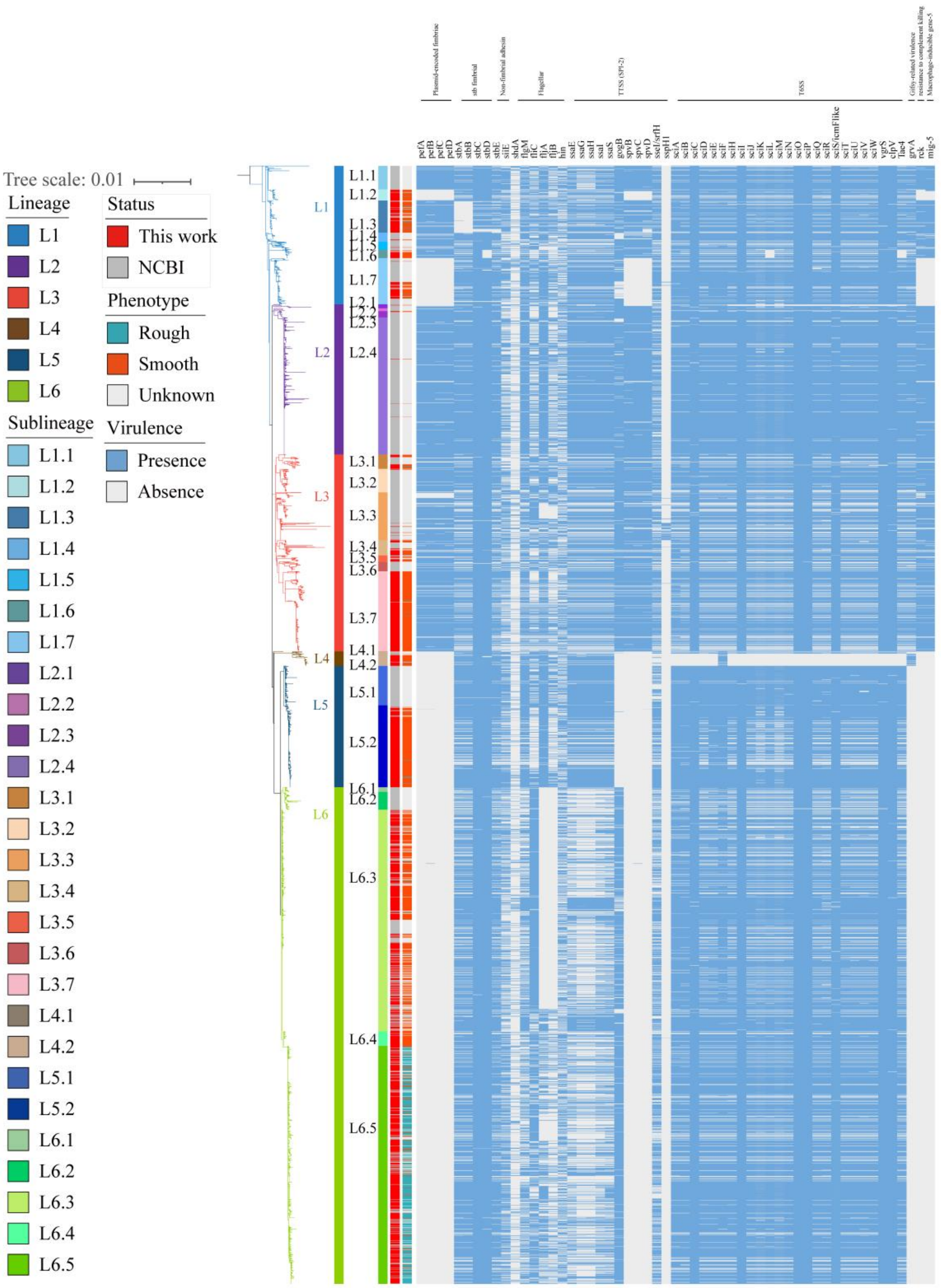

Figure S9. Maximum likelihood phylogeny of the global *S*. Typhimurium isolates with a heatmap showing the distribution of virulence factors.

The bands to the left of the heatmap indicate the lineage, sublineage, source, and colony morphology of the strains. The presence of a virulence gene is indicated in blue, and the absence of a gene is indicated in gray. Source data are provided as a Source Data file.

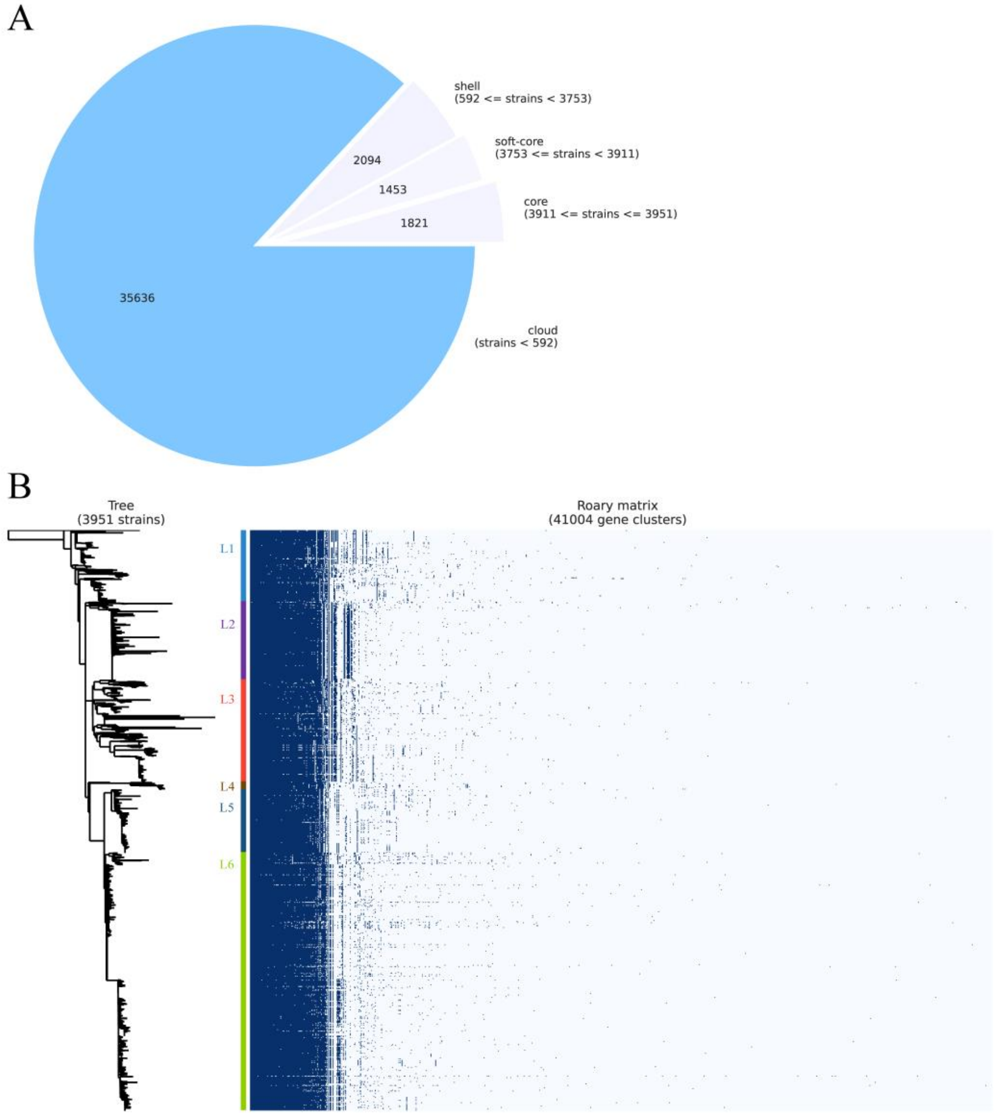

Figure S10. Pangenome analysis of the global *S*. Typhimurium isolates.

A total of 41,024 genes were identified by pangenome analysis based on the 3,951 isolates, including 1,831 core genes, 1,453 soft-core genes, 2,094 shell genes, and 35,636 cloud genes. **A** shows the proportions of core and accessory genes. **B** shows a heatmap displaying the distribution of the pangenome among isolates. Dark blue indicates the presence of a gene, and light blue indicates the absence of a gene. Source data are provided as a Source Data file.

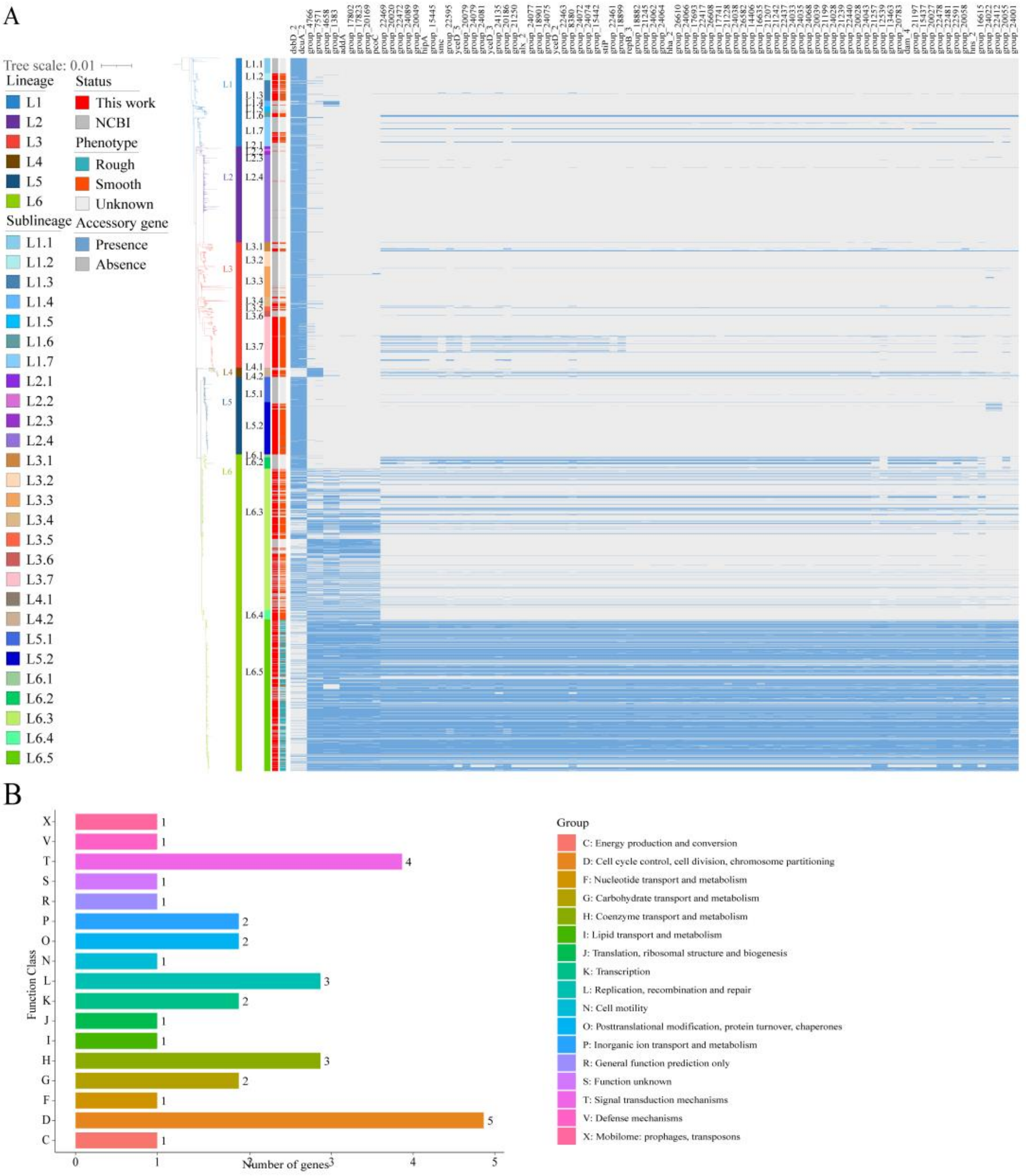

Figure S11. Accessory genes identified by pangenome analysis that might be associated with rough colony variants.

A shows the accessory genes significantly associated with the *S*. Typhimurium lineages, especially the rough colony variants. There were 89 specific genes screened by the chi-square test whose *P* value was lower than 1e-300. The presence of a gene is indicated in blue, and the absence of a gene is indicated in gray. **B** shows the COG functional classifications of significant accessory clusters of orthologous genes. COG functional classification showed that 29 genes mapped to 32 COG clusters, which are mainly involved in cell cycle control, cell division, and chromosome partitioning, signal transduction mechanisms, coenzyme transport and metabolism, and replication, recombination and repair. Source data are provided as a Source Data file. COG, Clusters of Orthologous Groups.

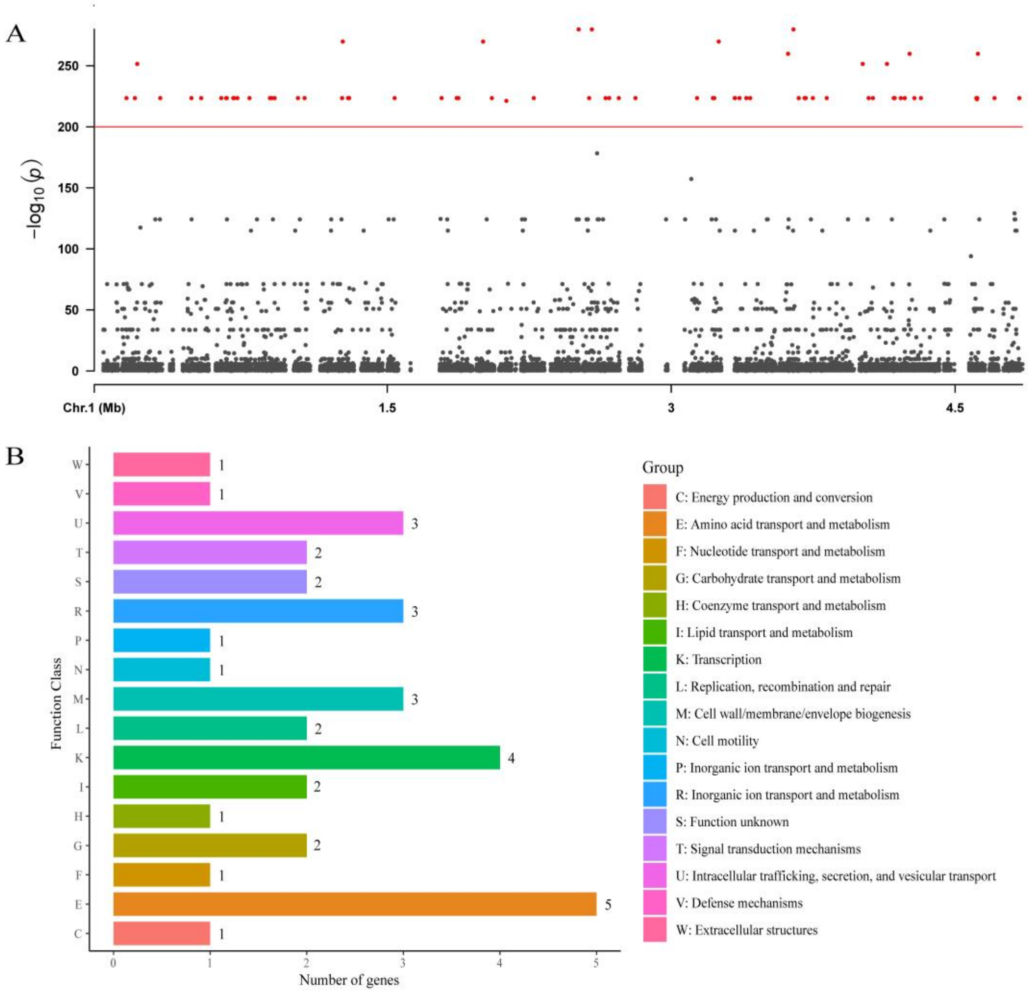

Figure S12. The SNPs identified by GWAS analysis that might be associated with rough colony variants.

A shows the SNPs identified by GWAS that are associated with the rough colony variants. There were 72 SNPs ($-\log_{10}(P) > 200$) identified to be significantly associated with rough colony variants; the red dots represent significantly associated SNPs. **B** shows the COG functional classifications of significantly associated SNPs. Among the significantly associated SNPs, 32 nonsynonymous mutations detected in 31 genes were mapped to 35 COG clusters, which were mainly associated with amino acid transport and metabolism, transcription, cell wall/membrane/envelope biogenesis, inorganic ion transport and metabolism, and intracellular trafficking, secretion and vesicular transport. Source data are provided as a Source Data file. SNP, single nucleotide polymorphism; GWAS, genome-wide association study; COG, Clusters of Orthologous Groups.

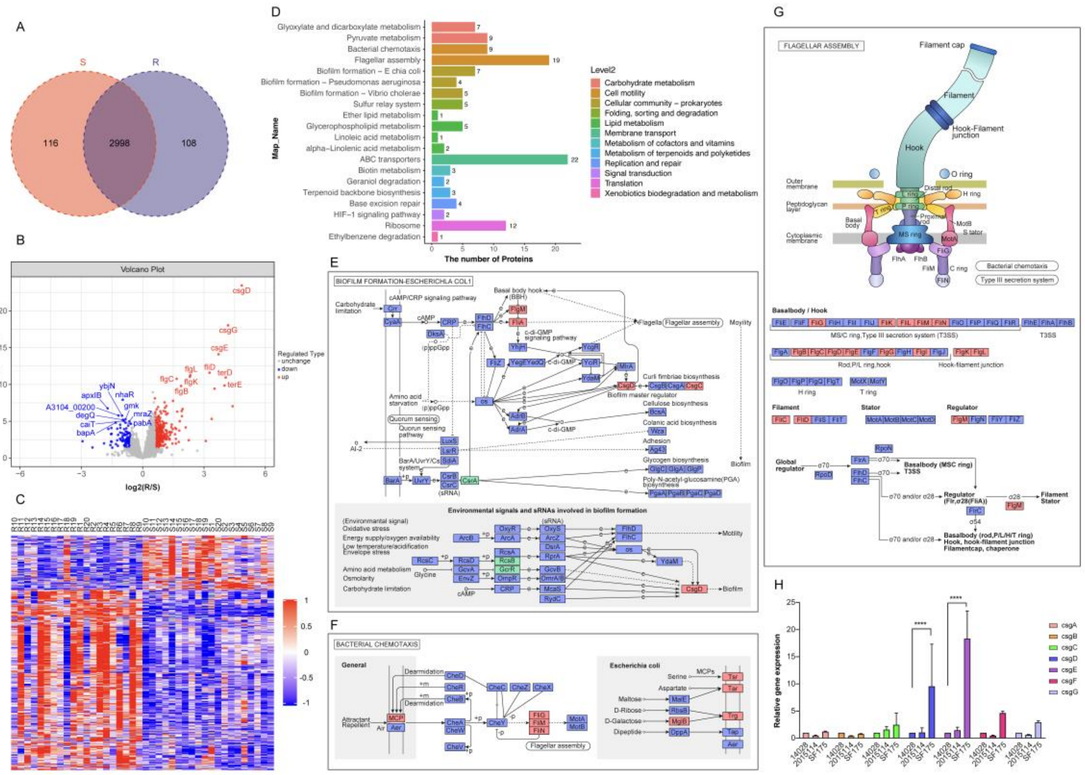

Figure S13. Proteomics analysis of the representative rough and smooth colony strains.

A. Venn diagram showing the overlap of the differentially regulated proteins among the rough and smooth colony strains. **B**. Volcano plot representing the differential proteins among the rough and smooth colony strains. Proteins were defined as differential if the fold change between the rough strains and smooth controls was > 1.5 or < 0.67 and the *P* value was < 0.05. The upregulated proteins (fold change > 1.5 and *P* value < 0.05) are shown in red, the downregulated proteins (fold change < 0.67 and *P* value < 0.05) are shown in green, and the unchanged proteins are shown in gray. The top 10 up- and downregulated proteins are marked. **C**. Hierarchical clustering heatmap showing the differential proteins among the rough and smooth colony strains. The up- and downregulated proteins are shown as red and green, respectively. **D**. Histogram of the KEGG pathways of the differential proteins. The top 20 KEGG pathways are displayed and mainly include the two-component system, ATP-binding cassette transporters, flagellar assembly, bacterial chemotaxis, quorum sensing, and biofilm formation pathways. **E**. KEGG pathway of biofilm formation. Boxes with a red background represent the upregulated protein, and boxes with a green background represent the downregulated protein. **F**. KEGG pathway of bacterial chemotaxis. **G**. KEGG pathway of flagellar assembly. **H**. The relative mRNA levels for the *csgBAC*-*csgDEFG* operons. Absolute qPCR values were normalized to the values for the bacterial 16S rRNA gene and the results are expressed as the fold change over the results from the reference strain ATCC 14028. Error bars represent standard deviations. Statistical significance was determined using a paired t-test (ns, not significant), and **** indicates the statistical significance of $p < 0.0001$. Source data are provided as a Source Data file. S, smooth; R, rough; KEGG, Kyoto Encyclopedia of Genes and Genomes; qPCR, quantitative polymerase chain reaction.

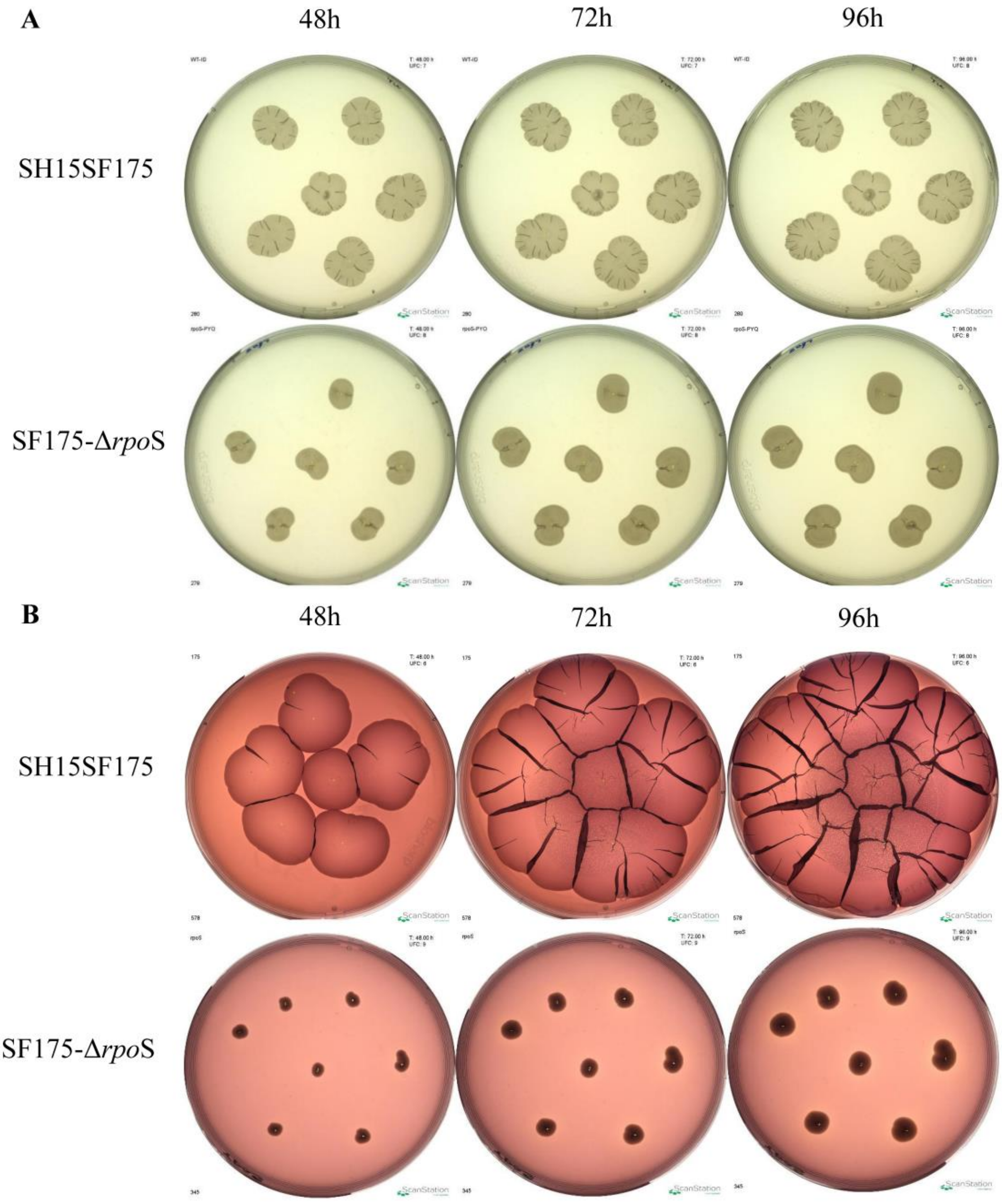

Figure S14. Growth of SF175 and SF175-Δ*rpoS* on different media.

A. Growth observed at 37 °C on the modified Swarmagar medium. **B.** Growth observed at 28 °C on the Congo Red medium. Source data are provided as a Source Data file.

SUPPLEMENTARY METHODS

Congo red assay

Bacteria were grown overnight in Luria-Bertani (LB) agar plates, and one colony of each strain was spotted onto LB agar without salt and supplemented with 40 μg/mL Congo red and 20 μg/mL Coomassie brilliant blue. The inoculated plates were incubated at 28 °C or 37 °C for seven days, and colonies were visualized every half hour with an automatic bacterial colony counter (ScanStation 100, Interscience, France).

Pellicle formation

The mat model was employed to quantify biofilm production at the air-liquid interface. *Salmonella* strains were cultivated in 3 mL of LB medium without NaCl in polystyrene round-bottom tubes at a temperature of 28 ℃ for a period of 5 days without agitation. To facilitate visual observation of the biofilm formed at the air-liquid interface, the liquid was carefully decanted and subsequently stained with a 1% solution of crystal violet.

Scanning electron microscopy (SEM) examination

A single colony was selected and inoculated at 28 °C or 37 °C for 24 hours. The prepared samples were then submitted for SEM analysis. Before SEM examination, coverslips and coupons were washed three times with sterile saline to remove loosely attached cells. The bacterial colony was then dipped into a PBS solution to ensure a consistent concentration of the bacterial suspension and fixed with a 2.5%

glutaraldehyde fixative (pH 7.2) overnight at 4 °C. Both the coverslips and coupons were washed three times with sterile distilled water followed by dehydration with a graded ethanol series (30%, 50%, 70%, 85%, 95%, and [3×] 100%). The coverslips and coupons were subsequently dried with a critical point dryer (Leica EM CPD 300) and sputter-coated with platinum with ion sputter (Hitachi E-1045). Finally, *Salmonella* morphotypes on the coverslips were analyzed with a scanning electron microscope (SU8010, Hitachi, Japan).

Crystal violet biofilm assays

Biofilm formation was assessed using the crystal violet assay[2]. Overnight cultures of the tested strains in LB medium without NaCl were added to 2 mL of API 0.85% NaCl, and the turbidity of each bacterial suspension was adjusted to 0.5 McFarland standard. Experiments were performed in 96-well polystyrene microtiter plates (Corning), and 20 μL of each 0.5 McFarland bacterial suspension was added to each well with 980 μL of LB medium, followed by 24 hours of incubation at 28 °C or 37 °C under static conditions. The total amount of biofilm biomass was quantified with crystal violet. After careful removal of the medium from the wells, the biofilms were washed three times with deionized water, and then 1 mL of 0.1% crystal violet solution was added to stain the biofilms for 20 minutes. Then, we gently aspirated the dye solution, each well was washed with sterile water three times and dried at room temperature, and 1.5 mL of 95% ethanol was added for destaining for 20 minutes. The OD_{600} of each well was measured three times with a microplate reader (BioTek, Winooski, VT, United States).

LB medium without NaCl was used as a negative control in all biofilm assays. All experiments were repeated three times.

Confocal laser scanning microscopy (CLSM) imaging

Single colonies after overnight culture were inoculated into 2 mL of API 0.85% NaCl, and the turbidity of each bacterial suspension was adjusted to 0.5 McFarland standard. Then, 100 μL of bacterial suspension was added to the laser confocal cell culture dish with 2 mL of LB without NaCl and incubated at 28 °C or 37 °C for 24 hours. The supernatant was removed, and the dishes were washed three times with phosphate-buffered saline (PBS) and then fixed with 2 mL of 2.5% glutaraldehyde for 1.5 hours. After aspirating the fixative and washing three times with PBS, each dish was stained with 2 mL of FITC-ConA (10 mg/L, green fluorescence, used to label extracellular polymeric substances [EPSs]) and PI (propidium iodide, 30 mg/mL, red fluorescence, used to label bacterial cells) solution in the dark at 4 °C for 30 minutes. Confocal microscopy images were obtained on Olympus FluoView FV3000 CLSM (FV3000, Olympus Corporation, Japan) with a 40X objective. The settings of the confocal microscope were as follows: the excitation/emission of PI and FITC-ConA were 535/615 and 550/570 nm, respectively, and the 3D architecture of the biofilm was scanned with a z-direction of 0.5 μm between each xy image. The confocal images were analyzed using COMSTAT software for the simultaneous visualization of bacterial cells and EPSs within intact biofilms.

Whole-genome sequencing and genome assembly

In this study, the genomes of 2,212 Chinese *Salmonella enterica* serovar Typhimurium (*S.* Typhimurium) isolates were sequenced. Genomic DNA from the Chinese isolates was prepared with the QIAamp DNA Mini Kit (Qiagen, Germany). Short-read sequencing was performed using the Illumina NovaSeq 6000 and MiSeq next-generation sequencing platforms as instructed by the manufacturer (Illumina, San Diego, CA, USA). The quality of the sequences was verified using Trimmomatic (v0.39)[3] and de novo assemblies of the genomes were obtained using SPAdes (v3.15.2)[4]. The 20 rough and 20 smooth colony strains selected were also submitted for long-read sequencing by the Nanopore PromethION platform in combination with the Illumina short-read sequencing platform (MiSeq). The long reads were first filtered for a minimum length of 1000 bp using Filtlong (v0.2.0) (https://github.com/rrwick/Filtlong) and then assembled using Flye[5]. The errors in the whole-genome sequences were corrected based on the Illumina sequencing data.

Single nucleotide polymorphism (SNP) calling and phylogenetic analysis

The genomes were mapped to the reference *S.* Typhimurium genome LT2 (GenBank accession NC_003197)[6] to identify the core genome SNPs using the Snippy (v4.6.0) pipeline (https://github.com/tseemann/snippy). SNPs were called using SAMtools (v1.12)[7] and FreeBayes (v 1.3.5)[8]. Recombinant regions were identified and removed using Gubbins (v 2.4.1)[9]. Core SNPs were identified using SNP-Sites (v 2.5.1)[10]. The maximum likelihood tree was inferred using IQ-TREE (v1.6.10)[11], a general time-

reversible (GTR) substitution model and gamma site heterogeneity model, and 1000 ultrafast bootstrap replicates were used. The genome sequence of *S*. Paratyphi A270 (accession number ERR326600) was used as an outgroup to identify the root and common ancestor of all *S*. Typhimurium strains[12]. The population structure was estimated using tree-independent hierarchical Bayesian clustering with hierBAPS (hierarchical Bayesian analysis of population structure) [13]. To infer the evolutionary dynamics of each branch, we selected 401 representative strains with definite isolation times and regions and used Bayesian Evolutionary Analysis Sampling Trees (BEAST2) to reconstruct the tree[14]. A Bayesian skyline model with a relaxed lognormal clock rate was chosen. Markov chain Monte Carlo (MCMC) generations were generated with one billion steps, and samples were taken at intervals of 1000 MCMC steps from six independent MCMC chains. Finally, we set a burn-in period of the first 10% steps in each chain and combined the log files of these chains before building the final maximum clade credibility (MCC) tree. Figtree was used to display the MCC tree produced by BEAST. Beautification of the phylogenetic tree and visualization of the associated data were carried out on the iTOL website (https://itol.embl.de/).

Plasmid detection

Complete plasmid sequences were generated from the 20 rough and 20 smooth colony strains sequenced by Nanopore long-read sequencing in combination with Illumina short-read sequencing. Plasmid sequence data were used for quality control and assembly and annotated using Prokka[15]. Plasflow (v 1.1.0)[16] was used to predict the

assembled plasmid genomes and plasmid sequence comparison and map generation were performed using BRIG[17] and Easyfig[18]. The locations of plasmid replicons, insertion sequences, antimicrobial resistance, and virulence factor genes were determined using PlasmidFinder[19], ISfinder[20], ResFinder[21], and the VFDB database[22], respectively.

Genome-wide association study (GWAS)

The Snippy pipeline was used to identify the SNPs in all of the assemblies. The file formats were converted using VCFtools (v 0.1.16)[23], and the Plink (v 1.07)[24] pipeline was used to calculate the *P* value of each SNP; *P* values below 1e-200 were considered significant. The qqman package (v 0.1.8) of R (v 3.5.1) was used to draw a Manhattan diagram. Clusters of Orthologous Groups (COG) classification of the genes identified by GWAS analysis was also performed by mapping to the COG database.

Recombination analysis

We performed recombination analysis using Gubbins software [doi: 10.1093/nar/gku1196] and genomic collinearity analysis using ACT software [doi: 10.1093/bioinformatics/bti553]. The results showed that the genomes of all these strains were highly similar and had no significant recombination.

Biofilm-related genes and mutation detection

We used Blast to detect biofilm formation-related genes in the assembled genomes, with the minimum coverage and minimum similarity threshold set to 80%. We used the

Snippy pipeline to identify the 755 bp intergenic region between the *csgBAC*-*csgDEFG* operon. We validated the sequence through Sanger sequencing.

Proteomic analysis of the rough and smooth colony strains

<u>Sample collection and preparation</u>

Forty strains of rough and smooth *Salmonella* typhimurium were inoculated onto the multiple soft agar medium using the inoculation loop method for preliminary identification of *Salmonella*. After incubating them at 37 ℃ for 18 hours, the colonies were gently scraped off with an inoculation ring, avoiding the culture medium components. The scraped colonies were transferred into 2 mL EP tubes containing 1.5 mL PBS. To obtain enough samples, this process was repeated until the liquid inside the EP tubes became visibly turbid. The EP tubes were labeled with the strain numbers on the walls or caps and centrifuged at 2,795 g (5,000 rpm) for 9 minutes. The bacterial samples were collected as sediments within the EP tubes.

The protein extraction process was conducted as follows[25]. Initially, three steel balls, each with a diameter of 3 mm, were mixed with an appropriate amount of quartz sand in a 2 ml thickened centrifuge tube, which was then placed on ice for later use. Thallus samples were collected and stored temporarily in a 2 ml screw-cap centrifuge tube at -80 ℃, with the remaining samples also stored at -80 ℃. Following this, 600 μL of lysate [SDT buffer (4% Sodium dodecyl sulfate, 100 mM Tris-HCl, 1 mM Dithiothreitol, pH 8.0)] was added, and the cap of the EP tube was tightly secured. The

sample then underwent a thorough freeze-grinding process. Ultrasonication was employed in intervals of 30 seconds, alternated with 30-second breaks, accumulating to a total duration of 40 minutes. Afterward, the sample was heated in a boiling water bath at 95 ℃ for 8 minutes. The sample was then centrifuged at 20 ℃ and 14,000 g for 30 minutes, after which the supernatant was removed and transferred to a new centrifuge tube.

Dithiothreitol (final concentration 10 mM) was added to each 100 μg of the above sample and mixed at 600 rpm for 1.5 hours at 37 °C. After cooling the samples to room temperature, Iodoacetamide (final concentration 20 mM) was added to block the reduced cysteine residues and incubated the samples for 30 minutes in the dark. The samples were transferred to filters (Microcon units, 10 kDa) and washed with 100 μl UA buffer (8M urea,150 mM Tris-HCl, pH 8.0) three times and then 100 μl 25 mM NH_4HCO_3 buffer twice. Trypsin (HLS TRY001C, No. 020201308) was added to the samples (trypsin: protein [wt/wt] ratio 1:50) and the samples were incubated at 37 ℃ for 15-18 hours (overnight). The resulting peptides were collected as a filtrate. The peptides of each sample were desalted on C18 Cartridges (Empore™ SPE Cartridges C18 [standard density], bed I.D. 7 mm, volume 3 ml, Sigma), concentrated by vacuum centrifugation and reconstituted in 40 μl of 0.1% (v/v) formic acid. The peptide content was estimated by UV light spectral density at 280 nm. For DIA experiments, iRT (indexed retention time) calibration peptides were spiked into the samples.

Data-dependent acquisition (DDA) mass spectrometry assay

The digested peptide pool was separated into 10 fractions using the Thermo Scientific™ Pierce™ High pH Reversed-Phase Peptide Fractionation Kit. Each fraction was desalted and reconstituted on C18 Cartridges (Empore™ SPE Cartridges C18 [standard density], bed I.D. 7 mm, volume 3 ml, Sigma) and then eluted in 40 μl of 0.1% (v/v) formic acid. Buffer A consisted of a 0.1% formic acid aqueous solution, with formic acid supplied by Fluka (565302-50mL-GL). The analytical gradient was set as follows: 0.00-3.00 minutes, Buffer B linearly increased from 5% to 8%; 3.00-43.00 minutes, Buffer B linearly increased from 8% to 26%; 43.00-48.00 minutes, Buffer B linearly increased from 26% to 40%; 48.00-50.00 minutes, Buffer B linearly increased from 40% to 90%; 50.00-60.00 minutes, Buffer B was maintained at 90%. iRT-Kits (Biognosys) peptides were spiked into the samples before DDA analysis. All sample fractions were analyzed on a TIMSTOF mass spectrometer (Bruker) connected to an Evosep One liquid chromatography system (Denmark). The mass spectrometer was operated in data-dependent mode for ion mobility-enhanced spectral library generation. The accumulation and ramp time were both set to 100 ms, capturing mass spectra within the m/z range of 100-1700 in positive electrospray mode, with a dynamic exclusion duration of 24.0 seconds. The ion source voltage was set to 1500 V, the temperature to 180 °C, and the dry gas flow to 3 L/min. Ion mobility scanning ranged from 0.75 to 1.35 Vs/cm^2, followed by eight cycles of PASEF MS/MS.

Mass spectrometry assay for data-independent acquisition (DIA)

Peptides from each sample were analyzed using a TIMSTOF mass spectrometer (Bruker) connected to an Evosep One liquid chromatography system (Denmark) in DIA mode. The mass spectrometer collected ion mobility MS spectra across a mass range of m/z 100-1700. Up to four windows were defined for single 100 ms TIMS scans according to the m/z-ion mobility plane. During PASEF MSMS scanning, the collision energy linearly increased as a function of mobility, from 20 eV at 1/K0 = 0.85 Vs/cm^2 to 59 eV at 1/K0 = 1.30 Vs/cm^2.

Mass spectrometry data analysis

For the analysis of DDA library data, the Spectronaut^{TM} 14.4.200727.47784 (Biognosys) software was used to search a FASTA sequence database, which was downloaded from the UniProt website (http://www.uniprot.org) on April 17, 2021. The iRT peptides sequence (Biognosys|iRT Kit|) was added to the database. The specific FASTA file of the sequence database was downloaded under the accession number uniprot_Salmonella_typhimurium_140545_0417 (20210417). Additionally, the database file has been uploaded and is available on iProX. Search parameters were set as follows: enzyme - trypsin; maximum missed cleavages - 1; fixed modification - carbamidomethyl(C); dynamic modifications - oxidation(M) and acetylation (Protein N-terminus). Data reporting for protein identification was based on 99% confidence, as determined by a false discovery rate (FDR) ≤ 1%.

DIA data was analyzed with Spectronaut^{TM} 14.4.200727.47784 by searching the

previously constructed spectral library. Key software parameters were set as follows: retention time prediction type - dynamic iRT; interference correction at MS2 level enabled; and cross-run normalization enabled. Results were filtered based on a Q value cutoff of 0.01 (equivalent to FDR < 1%). The proteomics methodologies were provided by Shanghai Applied Protein Technology Co., Ltd.

Validation of proteomic differential proteins

According to the proteomic results, the significantly differential proteins, including the *csg* operons *csgBAC* and *csgDEFG*, were selected to detect their mRNA abundance through real-time quantitative polymerase chain reaction (RT-qPCR) assays. The primer sequences used for RT-qPCR are shown in Supplementary Data 9. Total RNA was extracted using an RNA prep Cell/Bacteria Kit (Tiangen, Beijing, China), and the one-step TB Green® PrimeScript™ RT-PCR Kit II (Perfect Real Time; TaKaRa, Dalian, China) was used to perform RT-qPCR. The rough colony strain SF175, smooth colony strain 2015114, and control strain ATCC 14028 were selected as representatives, and the experiments were repeated three times for each sample.

Construction of the mutants with *csgD* gene knockout and a single nucleotide replacement (-44 T>G) in its promoter

To determine the possible role of the *csgD* gene and its promoter in mediating morphotype conversion and biofilm formation of the mrdar variants, a mutant with the knockout of the *csgD* gene and a mutant with a single nucleotide replacement (-44 T>G)

in its promoter were constructed for the rough colony strain SF175. The *csgD* gene knockout mutant was constructed by Ubigene Biosciences Co., Ltd. (Guangzhou, China) using a CRISPR-B^{TM} genomic editing vector[26-28]. The sgRNAs were designed on the CRISPR website (http://crispor.tefor.net/), and the primers and sgRNAs used in this study are listed in Supplementary Data 9. The plasmid and *csgD* donor were transferred to the wild-type rough colony strain SF175, and mutants were selected on LB agar plates with chloramphenicol and verified by PCR amplification and DNA sequencing (Supplementary Data 9). Moreover, a single point mutant with -44T>G substitution in the promoter of *csgD* was constructed by Micro Foresight Biosciences Co., Ltd. (Tianjin, China) and utilizing the pH73sacB suicide vector with the primers listed in Supplementary Data 9. Briefly, PCR was used to amplify the promoter region (-44T>G substitution) of the *csgD* gene with the primers A5967C-up-F and A5967C-down-R (Supplementary Data 9). The PCR products were then ligated into the pH73sacB suicide plasmid. The mutants were identified by screening transformants in the wild-type strain SF175 on LB agar plates containing 10% sucrose and 34 μg/ml chloramphenicol, and then verified by PCR amplification and Sanger sequencing (Supplementary Data 9). The phenotypic characteristics of the constructed *S.* Typhimurium mutants, including colony morphology, biofilm formation ability, and mRNA levels of *csg* operons, were verified according to the above-mentioned methods.

SUPPLEMENTARY REFERENCES

1 Mather, A. E. *et al.* Distinguishable epidemics of multidrug-resistant Salmonella Typhimurium DT104 in different hosts. *Science* **341**, 1514-1517 (2013). https://doi.org:10.1126/science.1240578

2 Baugh, S., Ekanayaka, A. S., Piddock, L. J. & Webber, M. A. Loss of or inhibition of all multidrug resistance efflux pumps of Salmonella enterica serovar Typhimurium results in impaired ability to form a biofilm. *J Antimicrob Chemother* **67**, 2409-2417 (2012). https://doi.org:10.1093/jac/dks228

3 Bolger, A. M., Lohse, M. & Usadel, B. Trimmomatic: a flexible trimmer for Illumina sequence data. *Bioinformatics* **30**, 2114-2120 (2014). https://doi.org:10.1093/bioinformatics/btu170

4 Bankevich, A. *et al.* SPAdes: a new genome assembly algorithm and its applications to single-cell sequencing. *Journal of computational biology : a journal of computational molecular cell biology* **19**, 455-477 (2012). https://doi.org:10.1089/cmb.2012.0021

5 Kolmogorov, M., Yuan, J., Lin, Y. & Pevzner, P. A. Assembly of long, error-prone reads using repeat graphs. *Nat Biotechnol* **37**, 540-546 (2019). https://doi.org:10.1038/s41587-019-0072-8

6 McClelland, M. *et al.* Complete genome sequence of Salmonella enterica serovar Typhimurium LT2. *Nature* **413**, 852-856 (2001). https://doi.org:10.1038/35101614

7 Li, H. *et al.* The Sequence Alignment/Map format and SAMtools. *Bioinformatics* **25**, 2078-2079 (2009). https://doi.org:10.1093/bioinformatics/btp352

8 Garrison, E. P. & Marth, G. T. Haplotype-based variant detection from short-read sequencing. *arXiv: Genomics* (2012).

9 Croucher, N. J. *et al.* Rapid phylogenetic analysis of large samples of recombinant bacterial whole genome sequences using Gubbins. *Nucleic Acids Res* **43**, e15 (2015). https://doi.org:10.1093/nar/gku1196

10 Page, A. J. *et al.* SNP-sites: rapid efficient extraction of SNPs from multi-FASTA alignments. *Microb Genom* **2**, e000056 (2016). https://doi.org:10.1099/mgen.0.000056

11 Nguyen, L. T., Schmidt, H. A., von Haeseler, A. & Minh, B. Q. IQ-TREE: a fast and effective stochastic algorithm for estimating maximum-likelihood phylogenies. *Mol Biol Evol* **32**, 268-274 (2015). https://doi.org:10.1093/molbev/msu300

12 Van Puyvelde, S. *et al.* An African Salmonella Typhimurium ST313 sublineage with extensive drug-resistance and signatures of host adaptation. *Nat Commun* **10**, 4280 (2019). https://doi.org:10.1038/s41467-019-11844-z

13 Cheng, L., Connor, T. R., Siren, J., Aanensen, D. M. & Corander, J. Hierarchical and spatially explicit clustering of DNA sequences with BAPS software. *Mol Biol Evol* **30**, 1224-1228 (2013). https://doi.org:10.1093/molbev/mst028

14 Bouckaert, R. *et al.* BEAST 2.5: An advanced software platform for Bayesian evolutionary analysis. *PLoS computational biology* **15**, e1006650 (2019). https://doi.org:10.1371/journal.pcbi.1006650

15 Seemann, T. Prokka: rapid prokaryotic genome annotation. *Bioinformatics* **30**, 2068-2069 (2014). https://doi.org:10.1093/bioinformatics/btu153

16 Krawczyk, P. S., Lipinski, L. & Dziembowski, A. PlasFlow: predicting plasmid sequences in metagenomic data using genome signatures. *Nucleic Acids Res* **46**, e35 (2018). https://doi.org:10.1093/nar/gkx1321

17 Alikhan, N. F., Petty, N. K., Ben Zakour, N. L. & Beatson, S. A. BLAST Ring Image Generator (BRIG): simple prokaryote genome comparisons. *BMC Genomics* **12**, 402 (2011). https://doi.org:10.1186/1471-2164-12-402

18 Sullivan, M. J., Petty, N. K. & Beatson, S. A. Easyfig: a genome comparison visualizer. *Bioinformatics* **27**, 1009-1010 (2011). https://doi.org:10.1093/bioinformatics/btr039

19 Carattoli, A. & Hasman, H. PlasmidFinder and In Silico pMLST: Identification and Typing of Plasmid Replicons in Whole-Genome Sequencing (WGS). *Methods Mol Biol* **2075**, 285-294 (2020). https://doi.org:10.1007/978-1-4939-9877-7_20

20 Siguier, P., Perochon, J., Lestrade, L., Mahillon, J. & Chandler, M. ISfinder: the reference centre for bacterial insertion sequences. *Nucleic Acids Res* **34**, D32-36 (2006). https://doi.org:10.1093/nar/gkj014

21 Zankari, E. *et al.* Identification of acquired antimicrobial resistance genes. *J Antimicrob Chemother* **67**, 2640-2644 (2012). https://doi.org:10.1093/jac/dks261

22 Chen, L. *et al.* VFDB: a reference database for bacterial virulence factors. *Nucleic Acids Res* **33**, D325-328 (2005). https://doi.org:10.1093/nar/gki008

23 Danecek, P. *et al.* The variant call format and VCFtools. *Bioinformatics* **27**, 2156-2158 (2011). https://doi.org:10.1093/bioinformatics/btr330

24 Purcell, S. *et al.* PLINK: a tool set for whole-genome association and population-based linkage analyses. *Am J Hum Genet* **81**, 559-575 (2007). https://doi.org:10.1086/519795

25 Wiśniewski, J. R., Zougman, A., Nagaraj, N. & Mann, M. Universal sample preparation method for proteome analysis. *Nat Methods* **6**, 359-362 (2009). https://doi.org:10.1038/nmeth.1322

26 Tang, X. *et al.* Structural basis for bacterial lipoprotein relocation by the transporter LolCDE. *Nature structural & molecular biology* **28**, 347-355 (2021). https://doi.org:10.1038/s41594-021-00573-x

27 Yu, Z. *et al.* Membrane translocation process revealed by in situ structures of type II secretion system secretins. *Nat Commun* **14**, 4025 (2023). https://doi.org:10.1038/s41467-023-39583-2

28 Hu, J. *et al.* Dietary D-xylose promotes intestinal health by inducing phage production in Escherichia coli. *NPJ Biofilms Microbiomes* **9**, 79 (2023). https://doi.org:10.1038/s41522-023-00445-w
