# Supplementary material for: Characterization of a Salmonella enterica serovar Typhimurium lineage with rough colony morphology and multidrug resistance
Source: Nat Commun. 2024 Jul 20;15:6123. doi: 10.1038/s41467-024-50331-y (PMC11271444; doi:10.1038/s41467-024-50331-y)
Supplement: Supplementary file 3 — Description of Additional Supplementary Files [file 41467_2024_50331_MOESM3_ESM.docx]

**Supplementary Data Legends**

File Name: Supplementary Data 1.

Description: The genome-sequenced *S*. Typhimurium isolates used in this study.

File Name: Supplementary Data 2.

Description: Distribution of the STs identified in this study among the *S*. Typhimurium isolates.

File Name: Supplementary Data 3.

Description: The accessory genes identified in this study that were associated with *S*. Typhimurium lineages.

File Name: Supplementary Data 4.

Description: The SNPs identified by GWAS analysis that were associated with the rough colony variants of *S*. Typhimurium.

File Name: Supplementary Data 5.

Description: The 20 rough and 20 smooth *S*. Typhimurium isolates selected as representatives in this study.

File Name: Supplementary Data 6.

Description: The proteins identified to be unique to rough and smooth colony variants.

File Name: Supplementary Data 7.

Description: The identified proteins and differential proteins among the rough and smooth colony variants.

File Name: Supplementary Data 8.

Description: GO and KEGG enrichment analysis.

File Name: Supplementary Data 9.

Description: The primers and sgRNAs used in this study.
