# Supplementary material for: Characterization of a Salmonella enterica serovar Typhimurium lineage with rough colony morphology and multidrug resistance
Source: Nat Commun. 2024 Jul 20;15:6123. doi: 10.1038/s41467-024-50331-y (PMC11271444; doi:10.1038/s41467-024-50331-y)
Supplement: Supplementary file 13 — Reporting Summary [file 41467_2024_50331_MOESM13_ESM.pdf]

nature portfolio

Corresponding author(s): Xuebin Xu, Juntao Yang, Hongbin Song, and Shaofu Qiu

Last updated by author(s): Jul 3, 2024

Reporting Summary

Nature Portfolio wishes to improve the reproducibility of the work that we publish. This form provides structure for consistency and transparency in reporting. For further information on Nature Portfolio policies, see our Editorial Policies and the Editorial Policy Checklist.

Statistics

For all statistical analyses, confirm that the following items are present in the figure legend, table legend, main text, or Methods section.

n/a	Confirmed	
☐	☒	The exact sample size (*n*) for each experimental group/condition, given as a discrete number and unit of measurement
☐	☒	A statement on whether measurements were taken from distinct samples or whether the same sample was measured repeatedly
☐	☒	The statistical test(s) used AND whether they are one- or two-sided<br>*Only common tests should be described solely by name; describe more complex techniques in the Methods section.*
☒	☐	A description of all covariates tested
☒	☐	A description of any assumptions or corrections, such as tests of normality and adjustment for multiple comparisons
☐	☒	A full description of the statistical parameters including central tendency (e.g. means) or other basic estimates (e.g. regression coefficient) AND variation (e.g. standard deviation) or associated estimates of uncertainty (e.g. confidence intervals)
☐	☒	For null hypothesis testing, the test statistic (e.g. *F*, *t*, *r*) with confidence intervals, effect sizes, degrees of freedom and *P* value noted<br>*Give P values as exact values whenever suitable.*
☐	☒	For Bayesian analysis, information on the choice of priors and Markov chain Monte Carlo settings
☒	☐	For hierarchical and complex designs, identification of the appropriate level for tests and full reporting of outcomes
☒	☐	Estimates of effect sizes (e.g. Cohen's *d*, Pearson's *r*), indicating how they were calculated

Our web collection on statistics for biologists contains articles on many of the points above.

Software and code

Policy information about availability of computer code

Data collection	Illumina Sequencing Platform (Hiseq, Miseq), Oxford Nanopore Sequencing platform (Min ION), and TIMSTOF mass spectrometer (Bruker).
Data analysis	Genomic comparisons were performed on the complete genomes from the 20 rough and 20 smooth colony strains to analyze their genetic differences in chromosomal regions such as single nucleotide polymorphisms (SNPs), indels, and rearrangements with BLAST (v 2.5.0), Snippy (v4.6.0) (https://github.com/tseemann/snippy), and Gubbins (v 2.4.1).<br>The core genome SNPs were identified using the Snippy (v4.6.0) pipeline, and the population structure was estimated using hierBAPS.<br>AMR genes were detected using ABRicate (https://github.com/tseemann/abricate) against the Comprehensive Antibiotic Resistance Database (CARD).<br>The virulence genes were aligned to the VF database (VFDB) using BLAST (v 2.5.0).<br>Known plasmid replicons were screened using ABRicate (https://github.com/tseemann/abricate).

For manuscripts utilizing custom algorithms or software that are central to the research but not yet described in published literature, software must be made available to editors and reviewers. We strongly encourage code deposition in a community repository (e.g. GitHub). See the Nature Portfolio guidelines for submitting code & software for further information.

Data

Policy information about availability of data

All manuscripts must include a data availability statement. This statement should provide the following information, where applicable:

- Accession codes, unique identifiers, or web links for publicly available datasets
- A description of any restrictions on data availability
- For clinical datasets or third party data, please ensure that the statement adheres to our policy

The publicly available sequences used in this study are available in GenBank (https://www.ncbi.nlm.nih.gov/datasets/genome/), with accession numbers listed in Supplementary Data 1. The sequencing data generated in this study have been deposited in the NCBI Sequence Read Archive under the BioProject number PRJNA981225 (https://www.ncbi.nlm.nih.gov/Traces/study/?acc=PRJNA981225). The annotated plasmid sequences have been deposited in GenBank under accession numbers PP852730 (https://www.ncbi.nlm.nih.gov/search/all/?term=pp852730) and PP852731 (https://www.ncbi.nlm.nih.gov/search/all/?term=pp852731). The mass spectrometry proteomics data have been deposited in the ProteomeXchange Consortium (https://proteomecentral.proteomexchange.org) via the iProX partner repository with the dataset identifier PXD047483 (https://proteomecentral.proteomexchange.org/cgi/GetDataset?ID=PXD047483). Source data are provided with this paper.

Research involving human participants, their data, or biological material

Policy information about studies with human participants or human data. See also policy information about sex, gender (identity/presentation), and sexual orientation and race, ethnicity and racism.

Reporting on sex and gender	Our study does not include sex- or gender-based analyses. Furthermore, there is no evidence that the strains in our study are associated with sex or gender.
Reporting on race, ethnicity, or other socially relevant groupings	Our study does not reporting on race, ethnicity, or other socially relevant groupings.
Population characteristics	Our study does not include population characteristics.
Recruitment	A total of 2212 strains of S. Typhimurium in China were investigated from our surveillance system. Due to the imperfection of the surveillance system, there may be selection bias, which makes the study imperfect.
Ethics oversight	The study was approved by the review board of the Chinese PLA CDC, Beijing, China. All patients provided informed consent for the collection of samples and relevant information.

Note that full information on the approval of the study protocol must also be provided in the manuscript.

Field-specific reporting

Please select the one below that is the best fit for your research. If you are not sure, read the appropriate sections before making your selection.

☒ Life sciences ☐ Behavioural & social sciences ☐ Ecological, evolutionary & environmental sciences

For a reference copy of the document with all sections, see nature.com/documents/nr-reporting-summary-flat.pdf

Life sciences study design

All studies must disclose on these points even when the disclosure is negative.

Sample size	We collected a diverse set of 2,212 Salmonella Typhimurium isolates from 20 different geographic regions of China between 2006 and 2018. From this larger sample, we randomly selected 20 representative rough colony strains and 20 representative smooth colony strains to assess their biofilm formation capabilities through crystal violet assays, pellicle formation assays, and confocal laser scanning microscopy imaging. This sample size of 40 strains was based on standard practices in the field and our goal of capturing the natural diversity within the larger isolate collection, as supported by the phylogenetic analysis we conducted.<br><br>For the multi-omics analyses, we again used this set of 40 representative strains. Finally, we randomly selected one strain from the 20 rough colony representatives, SH15SF175, to construct mutants with a csgD gene knockout and a single nucleotide promoter replacement.<br><br>The sample sizes used in this study were not determined through statistical calculations, as this is not a common practice in this type of exploratory microbiology research. However, the sample sizes are consistent with similar published studies in the field and are sufficient to provide reliable and representative insights into the diversity of biofilm formation phenotypes within the broader S. Typhimurium population.
Data exclusions	No strain was excluded.
Replication	All experiments were repeated twice or more, and reproduciblity was confirmed.
Randomization	The samples were not allocated into different groups in this study. Therefore, randomization is not relevant.

Blinding | In all experiments, investigators were blinded to minimize their impacts on experiments.

Reporting for specific materials, systems and methods

We require information from authors about some types of materials, experimental systems and methods used in many studies. Here, indicate whether each material, system or method listed is relevant to your study. If you are not sure if a list item applies to your research, read the appropriate section before selecting a response.

Materials & experimental systems

n/a	Involved in the study
☒	☐ Antibodies
☒	☐ Eukaryotic cell lines
☒	☐ Palaeontology and archaeology
☒	☐ Animals and other organisms
☒	☐ Clinical data
☒	☐ Dual use research of concern
☒	☐ Plants

Methods

n/a	Involved in the study
☒	☐ ChIP-seq
☒	☐ Flow cytometry
☒	☐ MRI-based neuroimaging

Plants

Seed stocks: Our study does not include plants.

Novel plant genotypes: Our study does not include plants.

Authentication: Our study does not include plants.
